# Brain connectivity patterns associated with duration of abstinence in methamphetamine use disorder

**DOI:** 10.1192/j.eurpsy.2026.10206

**Published:** 2026-07-31

**Authors:** G. Zhong, M. N. Potenza, J. Du, M. Zhao

**Affiliations:** 1 Shanghai Mental Health Center, Shanghai Jiao Tong University School of Medicine, Shanghai, China; 2 Department of Psychiatry and the Child Study Center, Yale School of Medicine, New Haven, CT, USA; the Connecticut Mental Health Center, New Haven, United States

## Abstract

**Introduction:**

Methamphetamine use disorder (MUD) represents a significant public health crisis characterized by neurobiological abnormalities. While neurofunctional variations across abstinence stages are documented, brain connectivity patterns associated with abstinence duration remain poorly characterized.

**Objectives:**

In this cross-sectional study, we characterized brain connectivity patterns associated with abstinence duration in MUD. We hypothesized that whole-brain functional connectivity patterns would covary with abstinence duration in MUD.

**Methods:**

In this cross-sectional study, we aimed to apply connectome-based predictive modeling (CPM) to resting-state functional magnetic resonance imaging (fMRI) data from MUD participants to identify abstinence-duration-associated connectivity patterns (Figure 1A). These patterns were validated in a separate, heterogeneous sample (Figure 1B). We subsequently conducted exploratory analyses to compare connectivity strength between MUD participants and healthy comparison subjects (HCs; Figure 1C) and examine connectivity variations across abstinence stages (<1 month, 1-3 months, 3-6 months, and 6-24 months) (Figure 1D).

**Results:**

Applying connectome-based predictive modeling with leave-one-out cross-validation to resting-state functional connectivity data from MUD participants stratified by abstinence duration (<1 month, 1-3 months, 3-6 months, 6-24 months; total *N*=85), we identified patterns significantly associated with abstinence duration (*r* = 0.51, *P* < .001) (Figure 2), validated in an independent cohort (*N*=48, *r* = 0.41, *P* < .004). These patterns comprised positive components showing strengthened within-network connectivity in motor/sensory, subcortical, and medial frontal networks, and enhanced between-network connectivity involving motor/sensory, cerebellum/brainstem, and subcortical networks; and negative components demonstrating reduced connectivity between motor/sensory and default mode networks, as well as among motor/sensory, medial frontal, and visual association networks (Figure 2). Exploratory analyses revealed systematic variation in strength, with healthy comparison subjects exhibiting intermediate connectivity relative to short-term (<1 month) versus prolonged (6–24 months) abstinent MUD individuals (Figure 3).

**Image 1:**
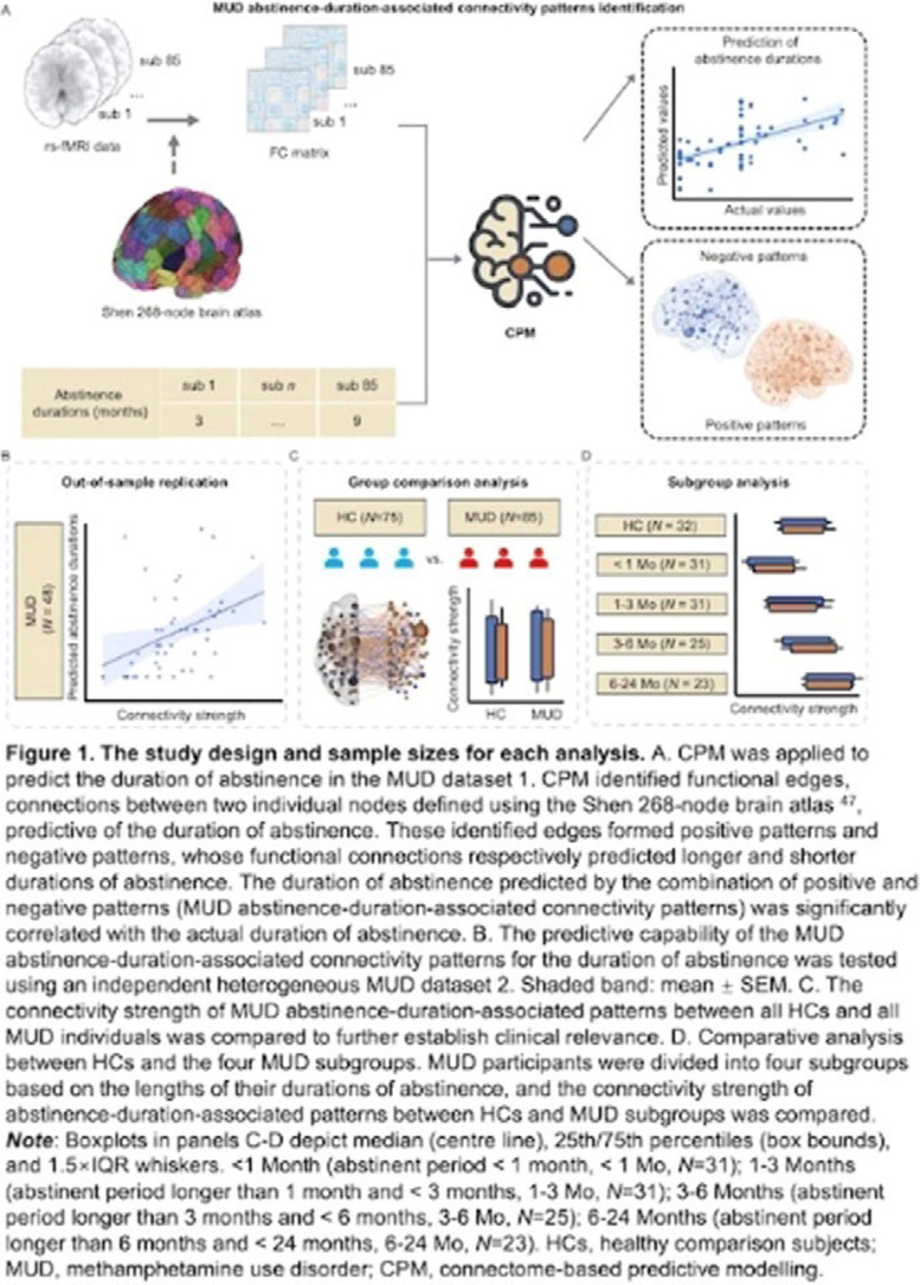
Image 1: Long description.

**Image 2:**
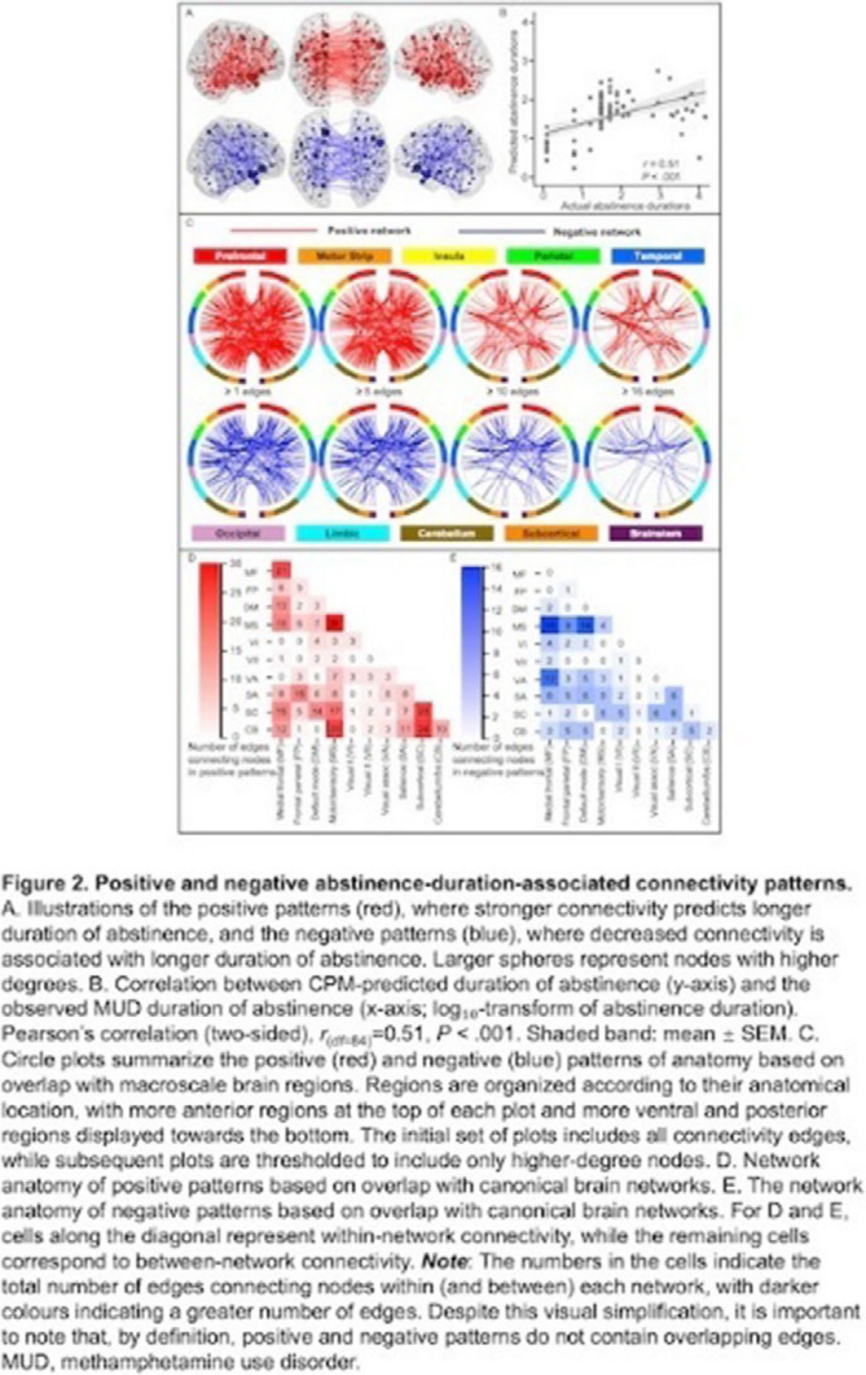
Image 2: Long description.

**Image 3:**
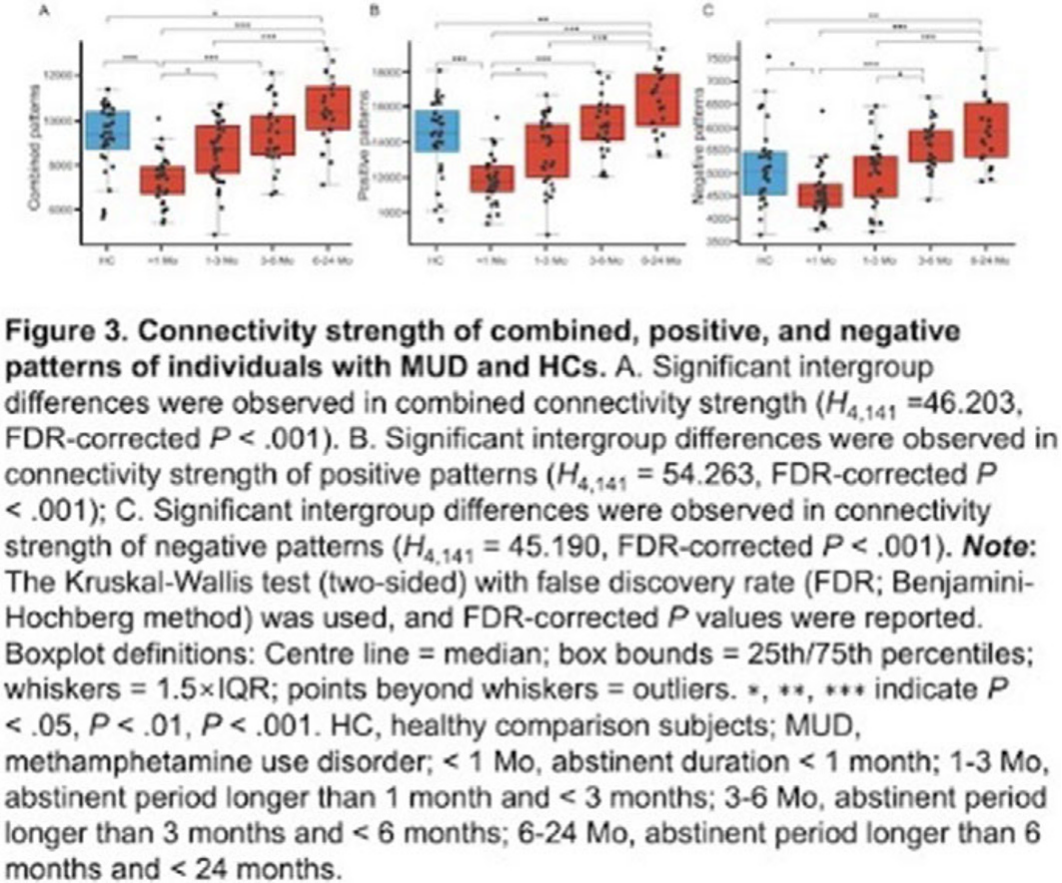
Image 3: Long description.

**Conclusions:**

In summary, this cross-sectional study identified abstinence-duration-associated connectivity patterns that covary with time abstinent from methamphetamine. Connectivity strength within these patterns demonstrated a significant association with abstinence duration across cohorts, revealing systematic variation along the abstinence continuum. These findings suggest dynamic neurofunctional reorganization during sustained abstinence from methamphetamine use.

**Disclosure of Interest:**

None Declared

## Long descriptions

### Image 1: Long description

Panel A shows the workflow for M U D abstinence-duration-associated connectivity patterns identification. It begins with r s - f M R I data and the Shen 268-node brain atlas, which are processed into F C matrices for 85 subjects. These matrices, along with abstinence durations in months, are fed into a C P M (connectome-based predictive modeling) process. The output includes a scatter plot showing a positive linear correlation between actual and predicted abstinence values, and brain maps showing negative and positive connectivity patterns.

Panel B, titled Out-of-sample replication, displays a scatter plot with a linear regression line and shaded confidence interval. The y-axis is predicted abstinence durations and the x-axis is connectivity strength for a dataset of 48 M U D subjects.

Panel C, titled Group comparison analysis, compares H C (N equals 75) and M U D (N equals 85). It includes a brain network diagram and a box plot showing connectivity strength. The box plots for H C and M U D show similar median values and distributions.

Panel D, titled Subgroup analysis, compares H C (N equals 32) with four M U D subgroups based on abstinence duration: less than 1 month (N equals 31), 1 to 3 months (N equals 31), 3 to 6 months (N equals 25), and 6 to 24 months (N equals 23). A series of horizontal box plots shows connectivity strength across these groups, with the M U D subgroups generally showing higher connectivity strength than the H C group.

Navigate back to Image 1:.

### Image 2: Long description

Panel A shows six brain models with red and blue nodes and edges. The top row displays positive patterns in red, and the bottom row displays negative patterns in blue. Panel B is a scatter plot with a linear regression line showing a positive correlation between actual abstinence duration on the x-axis and predicted abstinence duration on the y-axis. The correlation coefficient r equals 0.51 and P is less than .001. Panel C contains eight circular connectivity plots. The top row shows red positive networks and the bottom row shows blue negative networks. From left to right, the plots are thresholded by edge count: greater than 1, 5, 10, and 15 edges. Colored bars around the circles represent brain regions: Prefrontal, Motor Strip, Insula, Parietal, Temporal, Occipital, Limbic, Cerebellum, Subcortical, and Brainstem. Panels D and E are triangular heat map matrices. Panel D shows the number of edges connecting nodes in positive patterns, with values ranging from 0 to 30 in red. Panel E shows negative patterns with values ranging from 0 to 16 in blue. The axes for both matrices list brain networks including M F, F P, D M, M S, V I, V S, V A, S A, S C, and C B.

Navigate back to Image 2:.

### Image 3: Long description

The figure consists of three panels, A, B, and C, each containing a boxplot with individual data points overlaid. All panels share the same X-axis categories: H C, less than 1 Mo, 1 to 3 Mo, 3 to 6 Mo, and 6 to 24 Mo. The H C box is blue, while the four M U D groups are red.

Panel A: The Y-axis is Combined patterns ranging from 6000 to 12000. Connectivity strength drops significantly from H C to the less than 1 Mo group, then shows a linear increase across the subsequent abstinence periods, with the 6 to 24 Mo group reaching levels similar to or higher than H C.

Panel B: The Y-axis is Positive patterns ranging from 10000 to 18000. Similar to Panel A, there is an initial drop after H C followed by a steady upward trend as abstinence duration increases.

Panel C: The Y-axis is Negative patterns ranging from 3500 to 7500. The data follows the same trend as the previous panels, with the lowest connectivity in the less than 1 Mo group and a gradual recovery toward H C levels in the 6 to 24 Mo group.

Statistical brackets above the boxes in all panels indicate significant differences between groups, marked with asterisks representing P values less than .05, .01, or .001.

Navigate back to Image 3:.

